# Primary intracranial adenocarcinoma associated with an epidermoid cyst: a case report and literature review

**DOI:** 10.3389/fonc.2026.1780123

**Published:** 2026-04-20

**Authors:** Jiayi Lin, Jianhang Lin, Haiqing Xie, Liu Hu, Bing Liao, Shaolei Guo

**Affiliations:** 1 Department of Pathology, The First Affiliated Hospital, Sun Yat-sen University, Guangzhou, China; 2 Department of Neurosurgery, The First Affiliated Hospital, Sun Yat-sen University, Guangzhou, China

**Keywords:** adenocarcinoma, cerebellopontine angle, intracranial epidermoid cysts, intracranial tumor, malignant transformation

## Abstract

Intracranial epidermoid cysts are rare benign congenital lesions. Although malignant transformation is uncommon, most reported cases have involved squamous cell carcinoma. To our knowledge, transformation into adenocarcinoma has not previously been described. We present a 56-year-old man with a cerebellopontine angle lesion that demonstrated atypical contrast enhancement and mixed signals on SWI. The lesion was surgically resected, and postoperative histopathological examination revealed a poorly differentiated adenocarcinoma associated with a residual epidermoid cyst. Immunohistochemistry, including negative CK5/6 staining, excluded squamous cell carcinoma and common metastatic origins. Postoperative PET-CT showed no extracranial primary lesions. This appears to be the first well-documented case of primary intracranial adenocarcinoma associated with an intracranial epidermoid cyst. Careful radiologic evaluation and comprehensive immunohistochemical analysis are critical when atypical features are encountered. Further accumulation of similar cases is needed to better elucidate the mechanisms, biological behavior, and prognostic implications of this possible transformation process.

## Introduction

Intracranial epidermoid cysts are rare, benign congenital lesions, thought to arise from ectodermal inclusions during neural tube closure. They are most commonly located in the cerebellopontine angle (CPA) and parasellar region, accounting for approximately 1% of all intracranial tumors (1). Although histologically benign, epidermoid cysts are locally invasive and may encase or compress adjacent neurovascular structures, resulting in a range of neurological symptoms depending on their location and size (2).

Malignant transformation of intracranial epidermoid cysts is exceedingly rare, with the vast majority of reported cases progressing to squamous cell carcinoma (SCC) (3). This process is often associated with chronic inflammation, prior surgical intervention, or repeated cyst rupture, which may contribute to epithelial instability and carcinogenesis (4, 5). By contrast, adenocarcinomatous change appears to be exceptionally rare, and no well-documented cases of primary intracranial adenocarcinoma associated with an intracranial epidermoid cyst were identified in our review.

Herein, we present a rare case of a 56-year-old man with a lesion radiologically consistent with a CPA epidermoid cyst, which was histopathologically diagnosed as a poorly differentiated adenocarcinoma with residual epidermoid cyst. We further review the existing literature on malignant transformation of epidermoid cysts and discuss potential mechanisms, histopathological challenges, and diagnostic considerations.

## Case presentation

**Figure 1:**
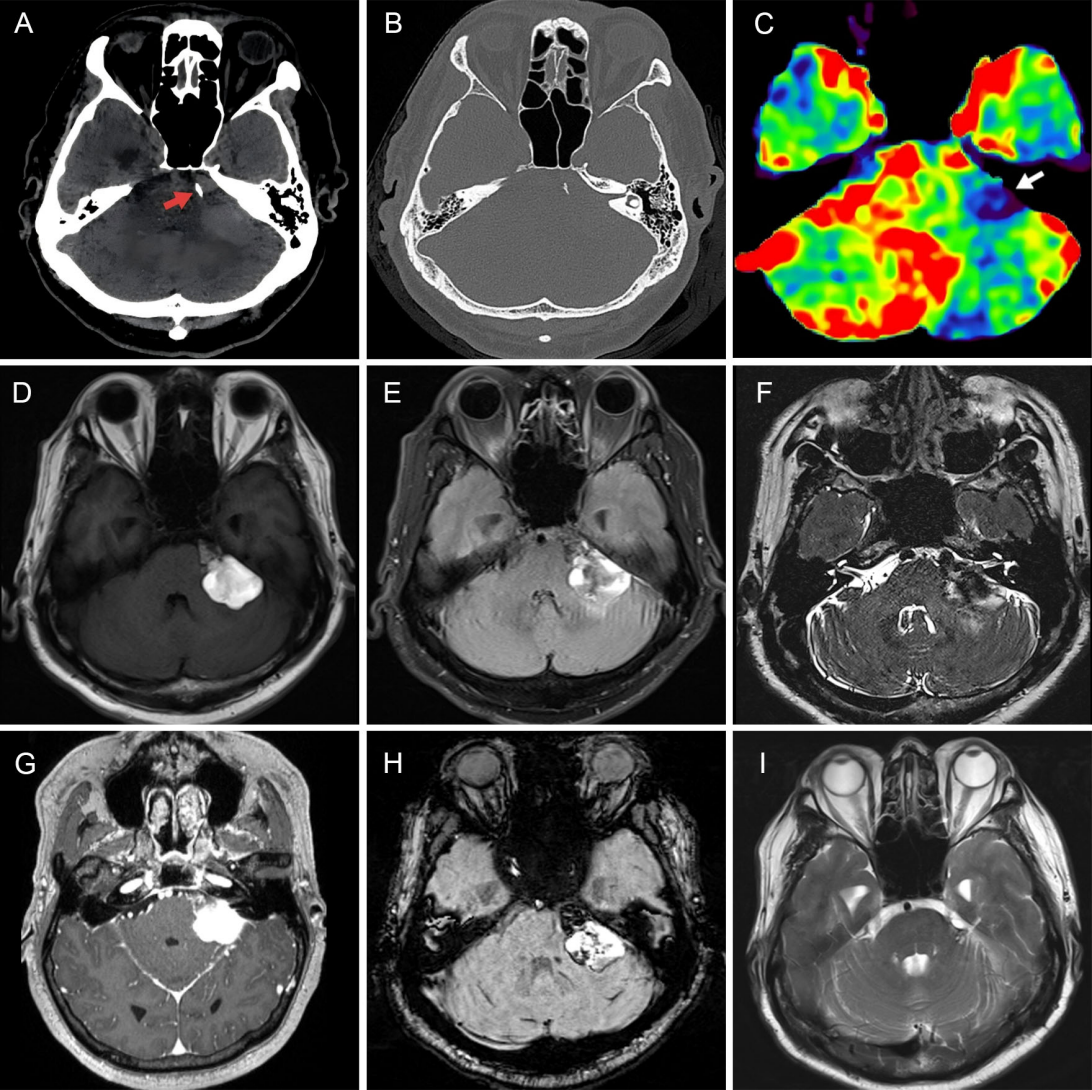
Preoperative and 8-month postoperative imaging. **(A, B)** Axial CT showing an irregular slightly hyperdense lesion with patchy calcifications in the left CPA (red arrows); no bony erosion. **(C)** CT perfusion indicating low perfusion in the lesion (white arrow). **(D–F)** MRI showing a 24 × 28 mm irregular CPA mass with hyperintensity on T1WI and mixed signals on T2-FLAIR and T2-SPACE. **(G)** Contrast-enhanced MRI demonstrating focal enhancement. **(H)** SWI revealing heterogeneous internal signals. **(I)** Follow-up MRI at 8 months confirming complete resection without recurrence.

A 56-year-old man without significant history of tumor presented with a 2-month history of progressive left-sided hearing loss and tinnitus, followed by dizziness, unsteady gait, and numbness over the left side of the face. Neurological examination revealed a positive Romberg sign. No other focal neurological deficits were noted. CT revealed an irregular, slightly hyperdense lesion in the left cerebellopontine angle, with patchy calcifications and no evidence of bony destruction (**Figures 1A, B**). CT perfusion (CTP) demonstrated predominantly low perfusion within the lesion (**Figure 1C**). MRI demonstrated a 24×28 mm mass in the left cerebellopontine angle, hyperintense on T1-weighted imaging and showed mixed high and low signal intensities on T2-FLAIR and T2-SPACE sequences (**Figures 1D–F**). Contrast-enhanced MRI revealed focal areas of marked enhancement within the lesion (**Figure 1G**). SWI demonstrated internal mixed signals suggestive of calcification or microhemorrhage (**Figure 1H**). The mass compressed cranial nerves VII and VIII and displaced the adjacent brainstem. Radiologically, a diagnosis of epidermoid cyst was favored.

**Figure 2:**
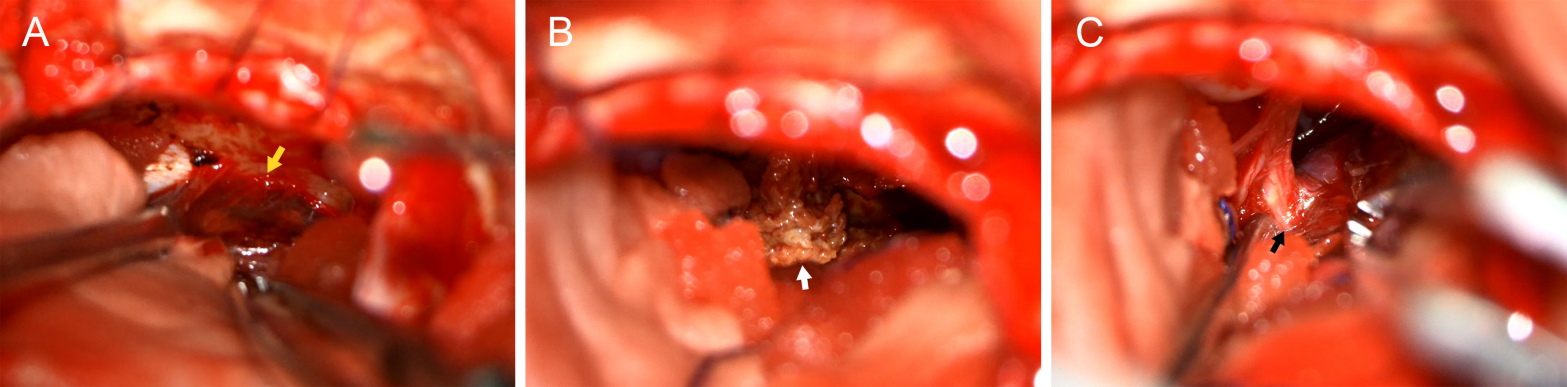
Intraoperative findings. **(A)** Cystic fluid with dark red, old hemorrhagic content (yellow arrow). **(B)** Grayish-white, cholesteatoma-like solid component (white arrow). **(C)** Tumor capsule adherent to cranial nerves VII and VIII (black arrow).

The patient subsequently underwent microsurgical resection via a retrosigmoid approach. Intraoperatively, the tumor was found to have both cystic and solid components. The cystic portion contained dark red, old hemorrhagic fluid, whereas the solid component consisted of grayish-white, cholesteatoma-like tissue (**Figures 2A, B**). The tumor capsule was tightly adherent to cranial nerves VII and VIII (**Figure 2C**). Intraoperative frozen section analysis showed edematous tissue with areas of collagenization, foamy cell formation, and hemosiderin deposition. A small number of epithelial cells and abundant eosinophilic keratinized material derived from squamous epithelium were noted. Some of the cells showed an increased nuclear/cytoplasmic ratio with atypia. The initial impression was consistent with an epidermoid cyst or epithelial neoplasm.

**Figure 3:**
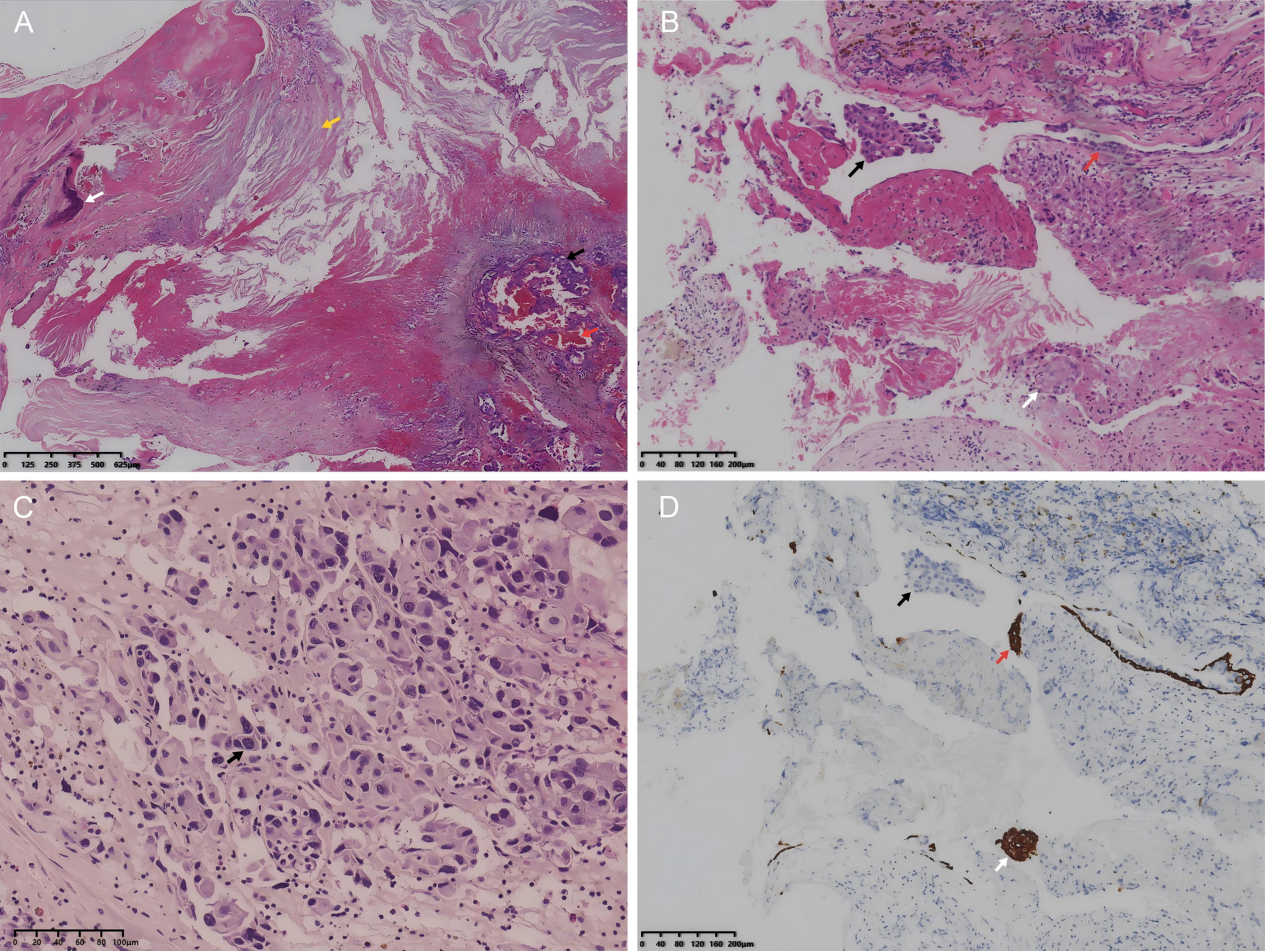
Hematoxylin and eosin (H&E) staining and CK5/6 immunohistochemistry. **(A)** H&E staining (×4) showing cyst wall and malignant tumor tissue (black arrow), with focal calcification (white arrow), hemorrhage (red arrow), and eosinophilic keratinous material in the lumen (yellow arrow). **(B)** H&E staining (×10) showing stratified squamous epithelium lining on the cyst wall (red arrow), a few shedded fragment of benign squamous epithelium (white arrow), and malignant tumor cells nest with obvious atypia (black arrow). **(C)** H&E staining (×20) showing tumor cells with marked nuclear atypia (black arrow). **(D)** CK5/6 immunohistochemistry (×10) showing diffuse cytoplasmic positivity in benign squamous epithelial cells (red and white arrows), while malignant tumor cells are negative (black arrow).

Subsequently, the surgical specimen was thoroughly sampled, fixed in formalin, and embedded in paraffin for sectioning. Histological examination revealed a cyst wall–like structure composed of fibrous tissue, with focal calcification, hemorrhage, and abundant eosinophilic keratinous material. Importantly, Carcinomatous tissue was observed infiltrating the cyst wall in a nested pattern (**Figure 3A**). The surface of the cyst wall was lined by stratified squamous epithelium. Within the cystic cavity, abundant eosinophilic keratinous material derived from squamous epithelium was present, along with a few shedded fragment of benign squamous epithelium and malignant tumor cell nests with obvious atypia (**Figure 3B**). In some tissue fragments, benign squamous epithelium and malignant adenocarcinoma components were identified simultaneously within the same cystic cavity. The malignant cells showed proliferation along the cyst lining, and in some areas, they appeared to extend directly beneath the benign squamous epithelium, accompanied by infiltrative growth into the deeper cyst wall. The malignant component showed diffuse infiltration, with tumor cells forming small nets and exhibiting an increased nuclear-to-cytoplasmic ratio, prominent nucleoli, and marked cellular atypia (**Figure 3C**). These findings support the coexistence of an epidermoid cyst and a malignant epithelial neoplasm.

**Figure 4:**
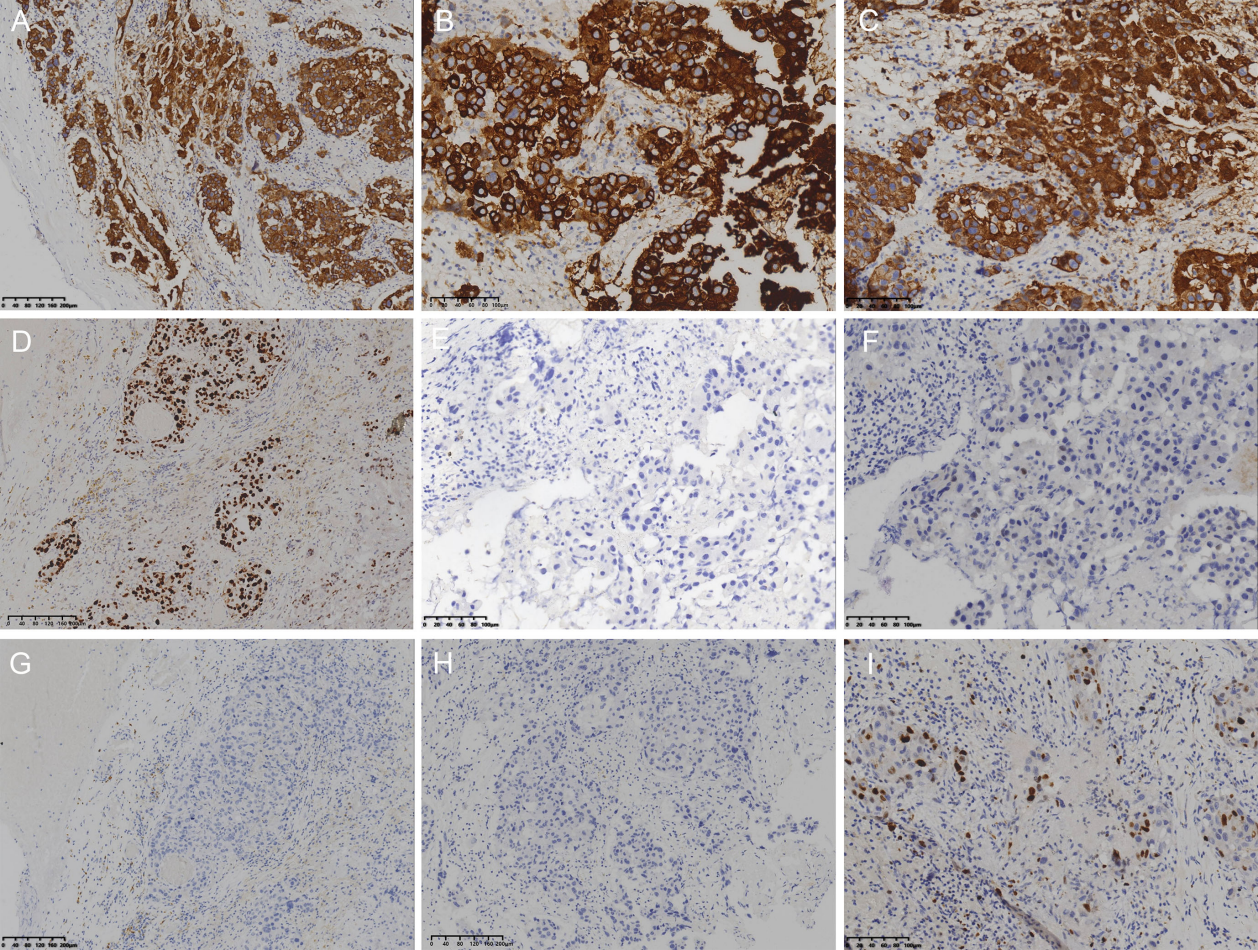
Immunohistochemical features. Tumor cells show strong cytoplasmic positivity for CK **(A)**, CK7 **(B)**, and CK19 **(C)**; diffuse nuclear positivity for P53 **(D)**; and are negative for TTF-1 **(E)**, CDX2 **(F)**, CK5/6 **(G)**, and P40 **(H)**. Ki-67 **(I)** staining shows a proliferation index of approximately 25%.

Immunohistochemically, the malignant tumor cells were strongly positive for CK (**Figure 4A**), CK7(**Figure 4B**), and CK19(**Figure 4C**) in the cytoplasm, indicating an adenocytic epithelial origin. P53 showed diffuse strong nuclear positivity, suggesting a possible TP53 gene mutation (**Figure 4D**). CK20 was focally positive. The malignant tumor cells were negative for TTF-1(**Figure 4E**), Napsin A, SATB2, CDX2(**Figure 4F**), NKX3.1, PSA, Synaptophysin, Chromogranin A, CK5/6(**Figure 4G**), and P40(**Figure 4H**), excluding pulmonary adenocarcinoma, gastrointestinal adenocarcinoma, prostatic adenocarcinoma, neuroendocrine neoplasm, squamous cell carcinoma and urothelial carcinoma origins. The benign squamous epithelium lining on the cyst wall or shedded within the cyst lumen were diffusely positive for CK5/6 in the cytoplasm (**Figure 3D**), whereas the malignant tumor cells were negative, further supporting adenocarcinomatous differentiation in a lesion associated with an epidermoid cyst. The Ki-67 proliferation index of the malignancy was estimated at 25% (**Figure 4I**), indicating moderate proliferative activity. These immunohistochemical results of the malignant tumor were consistent with a diagnosis of poorly differentiated adenocarcinoma.

Based on the hematoxylin-eosin morphology and immunohistochemical results, the findings support a diagnosis of an epidermoid cyst and a poorly differentiated adenocarcinoma. These findings support the possibility of malignant transformation in association with the epidermoid cyst.

A PET-CT scan performed immediately after surgery revealed no abnormal uptake in the lung, gastrointestinal tract, or other organs, further ruling out a metastatic origin. The patient subsequently underwent three courses of postoperative radiotherapy. At the 8-month follow-up, MRI demonstrated no evidence of local tumor recurrence (**Figure 1I**), and a follow-up PET-CT confirmed that there were no extracranial primary lesions or systemic disease. Taken together, the clinical history, imaging findings, histology, and immunohistochemistry support the diagnosis of a poorly differentiated adenocarcinoma associated with an epidermoid cyst and suggest a possible transformation process. The patient continues to undergo regular follow-up, with no evidence of extracranial malignancy identified to date.

## Discussion

A literature search of PubMed was conducted for articles published between January 2000 and April 2025 using the terms “intracranial epidermoid cyst” OR “intracranial epidermoid cysts” AND (“malignant transformation” OR “carcinoma” OR “adenocarcinoma”). Only publications reporting histopathologically confirmed malignant transformation of primary intracranial epidermoid cysts were included. Fifty-four articles were identified, and 63 eligible cases were included after screening. The reviewed cases showed that malignant transformation most commonly resulted in squamous cell carcinoma, whereas no well-documented case of primary intracranial adenocarcinoma arising from an intracranial epidermoid cyst was identified during the past 25 years.

Intracranial epidermoid cysts, while histologically benign, may cause considerable neurological morbidity due to their location and mass effect. Malignant transformation is exceedingly rare, with the majority of reported cases evolving into squamous cell carcinoma (SCC). Occasionally, undifferentiated carcinoma, poorly differentiated carcinoma, or sarcomatoid carcinoma have been documented (6–8). However, no well-documented case showing this pattern of adenocarcinomatous differentiation in association with an intracranial epidermoid cyst was identified in our review.

Our review of the literature over the past 25 years identified only a limited number of malignant transformation cases, predominantly involving SCC (**Table 1**). In contrast, the present case involved a cerebellopontine angle lesion was histopathologically diagnosed as a poorly differentiated adenocarcinoma. Residual benign squamous epithelium was observed within the lesion, suggesting a possible phenotypic transition from squamous to glandular differentiation. To our knowledge, this appears to be the first well-documented case of primary intracranial adenocarcinoma associated with an intracranial epidermoid cyst. This finding expands the recognized malignant potential of these lesions and suggests a more complex pathological evolution than previously appreciated.

**Table 1:** Summary of reported cases of malignant transformation of intracranial epidermoid cysts in the past 25 years.

Author(Year)	Age/Sex	Location	Histology	IHC profile
Lin et al. (2025) (23)	55/M	CPA	SCC	P63(+), P40(+), CK5/6(+), P53(+)
Elsarraj et al. (2025) (11)	61/M	CPA	SCC	P40+, Calretinin (-), SOX-10 (-)
Harary et al. (2025) (24)	55/F	CPA	SCC	PD-L1 CPS <1%
Alsadi et al. (2025) (25)	59/M	CPA	SCC	N/A
Yang et al. (2024) (18)	61/F​	Pre-pontine cistern	SCC	CK(AE1/AE3)(+), GATA3(+), Ki-67 20%​
Zhang et al. (2023) (26)	58/M	Right frontoparietal lobe, right lateral ventricle	SCC	N/A
Narasimhaiah et al. (2023) (5)	39/M	CPA	SCC	P63(+), MIB-1 40%
Eatz et al. (2023) (3)	67/F	CPA	SCC	N/A
Sakamoto et al. (2022) (19)	59/F	CPA	SCC	P53(+), P16(+)
Gabay et al. (2022) (27)	61/M	CPA	SCC	β-catenin (-), BRAF V600E(-)
Zuo et al. (2021) (28)	39/M	CPA	SCC	Not reported
	54/F	Suprasellar region	SCC	Ki-67 40%
	43/M	CPA	SCC	Ki-67 30%
	44/M	CPA	SCC	Ki-67 60%
	51/M	CPA	SCC	Ki-67 30%
	48/M	CPA	SCC	Ki-67 10%
	61/M	CPA	SCC	Ki-67 50%
	61/M	CPA	SCC	Ki-67 90%
	60/M	CPA	SCC	Ki-67 60%
Yanagawa et al. (2021) (7)	69/F	Caudal region of cerebellar vermis	Undifferentiated carcinoma	CK(+), P40 (-), CK5/6 (-), CDX-2 (-), TTF-1 (-), p53(+)
Demuth et al. (2019) (29)	67/F	CPA	SCC	N/A
Nagata et al. (2019) (30)	77/F	CPA	SCC	N/A
Fereydonyan et al. (2019) (31)	30/M	Left middle cerebellar peduncle	SCC	N/A
Cuoco et al. (2019) (32)	71/M	CPA	SCC	CK5/6(+)
Chen et al. (2019) (33)	43/M	CPA	SCC	CK(AE1/AE3) (+)
Liu et al. (2018) (34)	20/M	Left lateral ventricle	SCC	P63(+), P40(+), CK5/6(+), EMA(+), Pan-CK(+), P53 (-), GFAP (-), CK20 (-), Vimentin (-), S100 (-)
Suematsu et al. (2018) (35)	54/M	CPA	SCC	N/A
Badat et al. (2018) (36)	70/NA	CPA	SCC	P40(+), P16(+)
Ou et al. (2018) (37)	71/F	Right posterolateral cerebellar convexity	SCC	N/A
Solanki et al. (2017) (38)	47/F	CPA	SCC	N/A
Seif et al. (2017) (39)	83/M	Intracranial extradural, left posterior fossa	SCC	N/A
Roh et al. (2017) (40)	53/F	CPA	SCC	N/A
Ozutemiz et al. (2017) (20)	64/M	the posterior horn of the left lateral ventricle	SCC	N/A
Mascarenhas et al. (2017) (41)	35/F	CPA	SCC with areas of poorly differentiated sarcomatoid elements	CK5/6(+), P63(+), AE1/3(+), CK5/6(+) (focal), P63(+) (focal), CD34(-), S100(-), desmin(-), GFAP(-), CD31(-), myogenin(-)
Raheja et al. (2016) (42)	54/F	Brainstem, CPA	SCC	N/A
	37/F	Brainstem, CPA	infiltrative, poorly differentiated carcinoma most consistent with SCC	N/A
Pikis et al. (2016) (43)	77/M	CPA	SCC	N/A
Ding et al. (2016) (44)	55/F	Left temporal region and prepontine area	SCC	N/A
Chourmouzi et al. (2015) (45)	39/F	CPA	SCC	N/A
Vellutini et al. (2014) (46)	43/F	CPA	SCC	N/A
Kaplan et al. (2013) (10)	73/F	CPA	SCC with focal adenosquamous differentiation	N/A
Chon et al. (2012) (47)	43/M	CPA	SCC	pan-cytokeratin(+)
Raghunathan et al. (2011) (8)	60/M	CPA	SCC and Sarcomatoid Carcinoma	SCC component: CK5/6(+), pan-cytokeratin(+), EMA(+), P63(+), P53(+); Sarcomatoid component: Vimentin(+), SMA (+), focal MSA (+), P53(+)
Lakhdar et al. (2011) (48)	52/M	CPA	SCC	P53(+)
Kano et al. (2010) (49)	64/F	CPA	SCC	N/A
Hao et al. (2010) (50)	61/F	Right CPA, prepontine cistern, temporoparietal lobe	SCC	N/A
Ge et al. (2009) (51)	50/M	Right temporal lobe	SCC	N/A
Kim et al. (2008) (12)	72/F	Brain stem	SCC	N/A
Agarwal et al. (2007) (52)	45/M	Posterior fossa midline	SCC	CK(AE1/AE3) (+), MIB-1 index 42%
Kodama et al. (2007) (53)	67/M	CPA	poorly differentiated SCC	N/A
Pagni et al. (2007) (54)	65/F	Pineal region	SCC	N/A
Tamura et al. (2006) (55)	56/F	CPA	SCC	CK7 (+), MIB-1 index 47.9%
Michael et al. (2005) (56)	45/M	CPA	SCC	CK6 (+), CK8 (+)
Guan et al. (2004) (57)	42/F	Left frontotemporal region	SCC	N/A
Kadashev et al. (2003) (58)	47/F	The third ventricle and medial portions of temporal lobes	SCC	N/A
Jain et al. (2003) (9)	5/F	Right posterior temporal lobe	Poorly differentiated adenosquamous carcinoma	CK7(+), CAM5.2(+), keratin903(focal +), CEA+, MIB-1 >90%
Akar et al. (2003) (59)	N/A	CPA	SCC	N/A
Link et al. (2002) (60)	57/F	CPA	SCC	N/A
Tsugu et al. (2001) (61)	47/M	Cerebellar vermis, left cerebellar hemisphere, cisterna magna	SCC	MIB-1 index 10.9%
Nawashiro et al. (2001) (62)	46/M	Left temporal lobe and basal cisterns	Recurrent intracranial epidermoid cyst with malignant transformation (carcinomatous component)	N/A
Khan et al. (2001) (63)	53/M	Cystic lesion anterior to pons	SCC	N/A
Asahi et al. (2001) (6)	55/F	CPA	Poorly differentiated carcinoma	EMA (focal +), Keratin (-), AFP (-), HCG (-), PLAP (-), GFAP (-), MIB-1 index 30%
Sawan et al. (2001) (64)	66/M	Front of the pons/brainstem	SCC	N/A

Histologically, the malignant components in this case consisted predominantly of poorly differentiated epithelial cells with marked nuclear atypia, prominent nucleoli, and moderate eosinophilic cytoplasm. Focal areas showed a mucinous background, and residual benign squamous epithelium was also observed, suggesting a possible malignant phenotypic transition from squamous to glandular differentiation. Immunohistochemically, the malignant tumor cells showed strong diffuse positivity for CK, CK7and CK19, focal positivity for CK20, and strong nuclear overexpression of P53. The Ki-67 proliferation index was estimated at 25%, indicating moderate proliferative activity. These findings support the diagnosis of a poorly differentiated adenocarcinoma.

Importantly, both CK5/6 and P40—markers commonly used to identify squamous differentiation—were negative, effectively excluding squamous cell carcinoma or urothelial carcinoma. Meanwhile, a neuroendocrine neoplasm was also excluded, as both synaptophysin and chromogranin A were negative. These findings further support an adenocarcinoma. Immunomarkers associated with lung adenocarcinoma (TTF-1, Napsin A), gastrointestinal origin (CDX2, SATB2), and prostate or breast origin (PSA, NKX3.1, GCDFP-15, ER, PR, AR) were all negative. The patient had no significant history of tumor, and postoperative PET-CT imaging showed no evidence of extracranial primary lesions, further supporting a primary intracranial tumor rather than metastatic disease.

Taken together, the clinical, radiological, histological, and immunophenotypic findings support the interpretation of a primary intracranial adenocarcinoma associated with a preexisting epidermoid cyst and are compatible with a possible malignant transformation process. The absence of detectable extracranial malignancy on PET-CT, combined with the lack of immunohistochemical evidence supporting common primary sites, further reinforces this interpretation. In this context, the coexistence of benign squamous epithelium and malignant glandular components may reflect a process of epithelial transformation.

In previously reported cases, malignant transformation of intracranial epidermoid cysts has most commonly occurred in the cerebellopontine angle or posterior fossa, with squamous cell carcinoma (SCC) being the predominant histological subtype. In a systematic review of 94 studies encompassing 135 cases, Eatz et al (3) found that the vast majority of malignancies arising from epidermoid cysts were classified as SCC. In most reports, the diagnosis relied primarily on hematoxylin and eosin (H&E) morphology and limited immunohistochemical (IHC) markers, without a comprehensive panel to definitively determine tumor origin. In addition to SCC, rare cases of adenosquamous carcinoma have been described. Jain et al. (9) reported a 5-year-old girl with a large temporal lobe mass histologically diagnosed as poorly differentiated adenosquamous carcinoma, which recurred despite radical resection, chemotherapy, and radiotherapy. Histopathologically, the tumor was predominantly squamous with limited glandular differentiation, suggesting malignant transformation within a preexisting epidermoid or dermoid cyst. These observations highlight that although SCC remains the predominant histological outcome of malignant transformation, glandular differentiation may represent an underrecognized but clinically important pathway of tumor evolution (10).

The mechanisms underlying malignant transformation in epidermoid cysts remain poorly understood, and current theories are largely extrapolated from pathological observations and analogous conditions. Chronic inflammation is considered a key contributing factor. Repeated rupture, infection, or long-standing residual cysts may trigger chronic inflammation, leading to epithelial instability and DNA damage, which in turn promotes dysplasia and malignant transformation (5, 11, 12). Glandular metaplasia of squamous epithelium under inflammatory stress has been observed in middle ear cholesteatomas and chronic suppurative otitis media (13); similar pathways are well described in Barrett’s esophagus and pancreatic intraductal neoplasms (14, 15). In our case, the histological presence of both squamous and glandular components suggests a possible phenotypic transition. Besides, immunohistochemical findings showed strong nuclear positivity for P53, indicating a potential TP53 mutation—an early and common event in epithelial tumorigenesis, associated with genomic instability and evasion of apoptosis (16). Moreover, hypointense foci on susceptibility-weighted imaging (SWI), suggestive of chronic microhemorrhage or calcification, imply a hypoxic and oxidative stress environment, which has been implicated in promoting pro-oncogenic signaling and genetic alterations (17). Taken together, chronic inflammation, molecular alterations such as TP53 mutation, and a hypoxic microenvironment may act synergistically in the rare malignant transformation of epidermoid cysts. Nevertheless, further studies are needed to validate these proposed mechanisms.

At the molecular level, comparative analysis of the squamous and adenocarcinoma components would be valuable for assessing their clonal relationship and further clarifying the mechanism of malignant transformation. In particular, next-generation sequencing (NGS) may help determine whether the two components share key genetic alterations, which would provide additional support for a common origin and evolutionary progression within the same lesion. Although such analysis was not performed in the present case, it represents an important direction for future investigation.

In terms of radiological features, distinguishing malignant transformation from a complicated but benign epidermoid cyst is particularly important. Malignant transformation is more likely to be associated with nodular or irregular contrast enhancement, development of a solid component, perilesional edema, and invasive involvement of adjacent neurovascular structures (18–20). In contrast, benign epidermoid cysts complicated by inflammation or hemorrhage may show signal heterogeneity or limited enhancement, but they usually lack a discrete enhancing solid component and other aggressive features (21, 22). In the present case, the lesion retained a typical cerebellopontine angle location for an epidermoid cyst; however, several atypical findings raised concern for a malignant process, including focal marked enhancement, mixed susceptibility signals suggestive of calcification or chronic hemorrhage, intraoperative identification of both cystic and solid components, and tight adherence to adjacent cranial nerves. These findings do not by themselves prove malignant transformation, but they are less typical of a benign complicated cyst and therefore support preoperative suspicion of an associated malignant process.

From a diagnostic perspective, this case expands the known spectrum of epidermoid cyst malignant transition, suggesting that transformation may not be confined to squamous differentiation but may also include glandular components. It highlights the importance of maintaining diagnostic vigilance even in radiologically typical lesions. Intraoperative findings such as abnormal tissue texture, significant cytologic atypia on frozen section, or atypical enhancement patterns should prompt a comprehensive IHC evaluation, and molecular analysis when necessary.

Ongoing documentation and systematic review of such rare cases are essential to deepen our understanding of the malignant transformation potential of epidermoid cysts. Establishing multi-institutional collaborative databases will further help delineate their pathological evolution and improve diagnostic and therapeutic strategies.

Several limitations should be acknowledged. First, although extensive clinical and radiological evaluation did not reveal any extracranial primary tumor, the possibility of an occult extracranial primary lesion cannot be entirely excluded. Second, molecular analysis was not performed to compare the genetic profiles of the squamous and adenocarcinoma components, which could have provided additional evidence regarding clonality and the mechanism of malignant transformation. Third, the relatively short follow-up limits long-term assessment.

## Conclusion

We report an exceedingly rare case of a poorly differentiated adenocarcinoma associated with an intracranial epidermoid cyst, with findings that support the possibility of malignant transformation. This case suggests that the malignant transformation potential of epidermoid cysts may not be limited to squamous differentiation and highlights the possibility of glandular transformation within these lesions. Our findings underscore the importance of maintaining suspicion for an associated malignant process when an epidermoid cyst shows atypical imaging or intraoperative features despite an otherwise benign radiological appearance. Comprehensive histopathological and immunohistochemical evaluation as well as postoperative PET-CT is essential for accurate diagnosis and for ruling out metastatic disease. Further accumulation of such rare cases is warranted to elucidate their underlying molecular mechanisms, clinical behavior, and prognostic characteristics.

## Data Availability

The original contributions presented in the study are included in the article/supplementary material. Further inquiries can be directed to the corresponding authors.
